# One-dimensional core–shell motif nanowires with chemically-bonded transition metal sulfide-carbon heterostructures for efficient sodium-ion storage†

**DOI:** 10.1039/d1sc04163k

**Published:** 2021-10-27

**Authors:** Pengcheng Wei, Jinliang Zhu, Yuyan Qiu, Guifang Wang, Xingtao Xu, Shaojian Ma, Pei Kang Shen, Xing-Long Wu, Yusuke Yamauchi

**Affiliations:** Guangxi Key Laboratory of Processing for Non-ferrous Metals and Featured Materials, School of Resources, Environment and Materials, Collaborative Innovation Center of Sustainable Energy Materials, Guangxi University Nanning 530004 P. R. China jlzhu85@163.com; International Center for Materials Nanoarchitectonics (WPI-MANA), National Institute for Materials Science 1-1 Namiki Tsukuba Ibaraki 305-0044 Japan XU.Xingtao@nims.go.jp; National & Local United Engineering Laboratory for Power Batteries, Faculty of Chemistry, Northeast Normal University Changchun Jilin 130024 P. R. China; School of Chemical Engineering and Australian Institute for Bioengineering and Nanotechnology (AIBN), The University of Queensland Brisbane QLD 4072 Australia y.yamauchi@uq.edu.au

## Abstract

Herein, a chemical-vapor deposition-like strategy was developed for the synthesis of versatile core–shell transition metal sulfide (TMS)@carbon nanowires with chemically-bonded heterostructures and significantly improved electrochemical performance. The morphological evolution observations revealed the simultaneous growth of TMS nanowires and their bonding with an ultrathin carbon layer. The resulting core–shell heterostructured nanowires possessed notable advantages, including fast ion/electron diffusion rates, improved conductivity, and chemical/mechanical stability, thereby leading to remarkable reversible capacity, rate capability, and cycling stability for Na-ion storage applications. The *in situ* transmission electron microscopy and *in situ* X-ray diffraction studies for FeS@C demonstrated the crystalline phase evolution between hexagonal and tetragonal FeS species during the electrochemical charging/discharging process, clearly indicating the excellent Na-ion storage performance of FeS@C nanowires. This work provides a new methodology for achieving 1D core–shell nanoarchitectures, while elucidating the electrochemical reaction mechanism underlying Na-ion storage in TMS materials.

## Abstract 2

A chemical-vapor deposition-like strategy was developed for the synthesis of versatile core–shell transition metal sulfide (TMS)@carbon heterostructured nanowires for electrochemical sodium ion storage.

## Graphical abstract

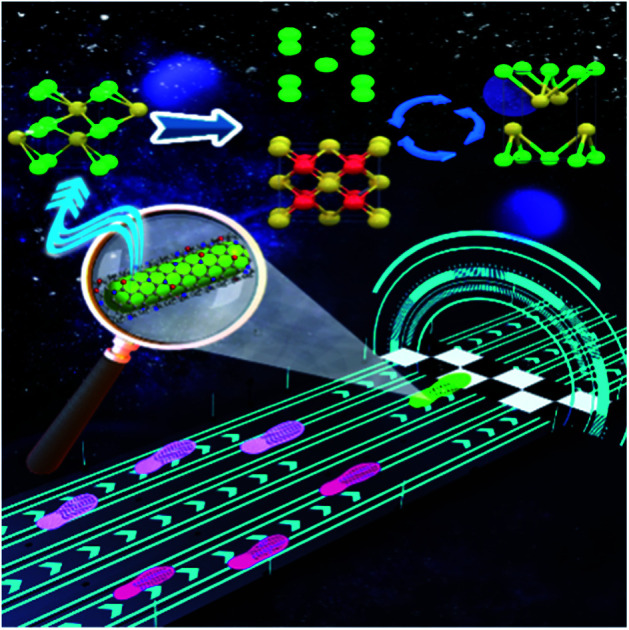

## Introduction

Owing to their superior physicochemical properties, transition metal sulfides (TMSs) are one of the most widely studied inorganic materials in various industrial fields relevant to secondary ion batteries,^1–3^ supercapacitors,^4,5^ and catalysts.^6^ In particular, TMSs have been considered as promising anode materials for sodium-ion batteries (SIBs).^7,8^ It has been demonstrated that the morphology and structure of TMS materials can significantly affect their sodium-ion storage behaviors,^9^ which has encouraged an increasing number of studies in the field of TMS nanoarchitectures,^10^ for designing, modifying, and functionalizing TMS nanomaterials at the atomic/molecular level. The past few decades have witnessed significant advancements in the synthesis and preparation of nanoarchitectured platforms with controllable dimensionality, including 0D nanodots/spheres, 1D nanotubes/wires/rods, and 2D nanosheets.^11–13^ Among them, 1D nanostructures have attracted notable attention due to their sophisticated nanoarchitectures.^11,14^

Generally, common TMS materials are characterized by limited electrical conductivity and poor chemical/mechanical stability in their bulk form, which not only impacts their electrochemical reaction performance, but can also result in severe volume changes during charge/discharge cycles, eventually compromising the electrode structure and causing the shedding of the active material.^15^ Although optimized single TMS nanostructures could improve the electrochemical performance of the electrode to some extent,^16^ this strategy cannot address all aspects of the challenges discussed above. In this regard, innovative strategies for achieving 1D core–shell heterostructures, especially 1D TMS@carbon heterostructured nanowires, are highly desired in the field of energy storage applications.^17^ However, to the best of our knowledge, this challenge has not been efficiently tackled to date.

In addition to hydrothermal/solvothermal strategies for general synthesis of TMS@carbon materials,^18–20^ TMS@carbon heterostructured materials, especially TMS nanowires coated with a uniform carbon layer, could also be obtained by other two strategies, including (i) the growth of the TMS core with further carbon coating, and (ii) the synthesis of the TMS precursor@carbon heterostructure with further sulfidizing treatment. Unfortunately, these two methods include complicated multistep reactions, require harsh experimental conditions, and might liberate toxic H_2_S gas,^21^ thus rendering the control of the nanostructure growth a dangerous and time-consuming process. In addition, the obtained materials usually exhibit a carbon coating with uneven thickness and weak bonding between the TMS and the carbon layer, which is unfavorable for charge-transfer applications and cannot totally guarantee the prevention of volume changes during the charge/discharge process.^20–22^ Recently, a simple one-step synthesis of Ni_3_S_2_ nanowires through low-temperature pyrolysis of a sulfonic acid group-containing resin with Ni foam was reported.^23^ Although some carbon species were also generated with the Ni_3_S_2_ nanowires, the obtained carbon could not be uniformly covered on the surface of nanowires. Therefore, obtaining uniform TMS@carbon heterostructures, especially 1D nanowires, through a facile, environmentally compatible, and universal process is still challenging at present and needs to be addressed as soon as possible.

In this study, we reported the fabrication of TMS@NSC (TM = Fe, Co, Cu; NSC = N,S-doped carbon) nanowires with core–shell 1D chemically-bonded heterostructured nanoarchitectures using a one-step chemical vapor deposition (CVD)-like strategy (Fig. S1†). Different from the previous studies, we introduced sulfur/nitrogen-containing solid precursors (thiourea formaldehyde resin) instead of conventional gaseous precursors to provide sulfur and carbon sources. The pyrolysis of this resin could generate a sulfur-/carbon-containing gas (*e.g.*, NH_2_CN, CS_2_, hydrocarbons, *etc.*),^24^ which could then react with metallic foam (Fe, Co, and Cu) to epitaxially grow 1D TMS nanowires; parallelly, an ultrathin carbon layer was formed on the grown TMS nanowires forming 1D core–shell heterostructure. The above two-fold process was defined as a “solid–gas–solid–liquid–solid” reaction mechanism, where “solid” refers to the solid resin, “gas–solid–liquid” corresponds to the continuous process including decomposition of the resin to a sulfur-/carbon-containing gas, and the reaction of the gaseous precursor with metallic foam to form carbon-dissolved TMS solid solution, and the final “solid” means the generation of the TMS@NSC heterostructure after the dissolved carbon concentration reaches saturation. Intriguingly, a strong C–S–TM bonding interaction was observed between the carbon shell and the TMS core, which would considerably benefit the reversible conversion reaction of TMS@NSC nanowires during the electrochemical process. As a proof of the concept, our FeS@NSC nanowires delivered excellent Na-ion storage performance in terms of reversible capacity, long-term cycling stability, and rate performance, thereby highlighting the significance of 1D core–shell heterostructure nanoarchitectures on modular nanomaterials with enhanced electrochemical performance.

## Results and discussion

**Fig. 1:**
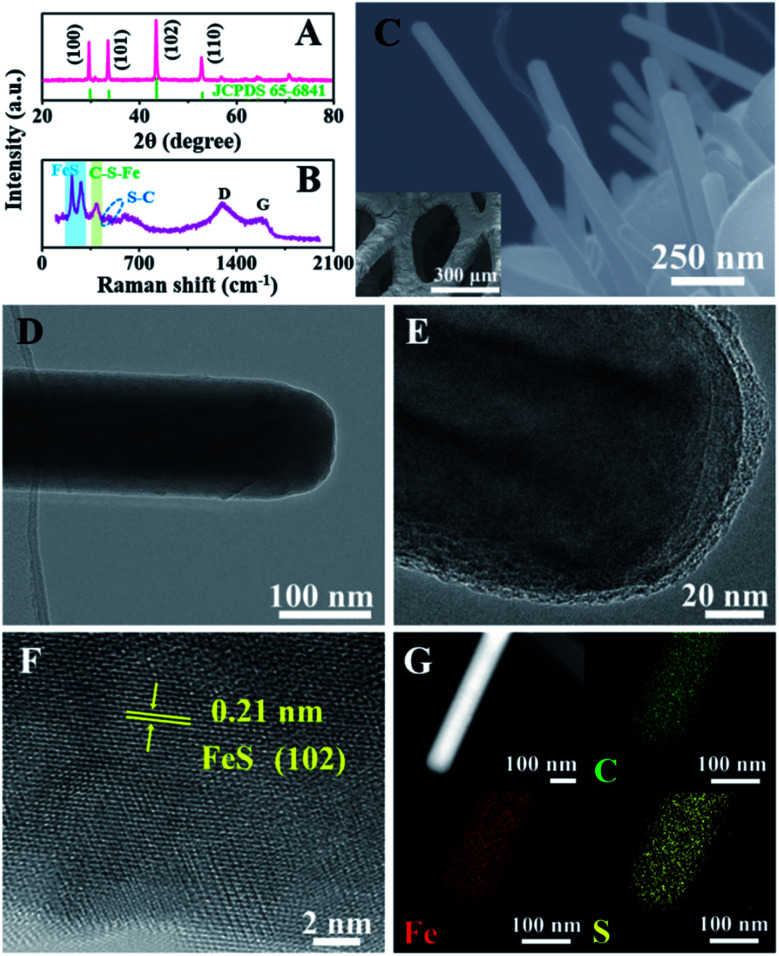
(A) XRD pattern, (B) Raman spectrum, (C) SEM, (D and E) TEM, (F) HRTEM, and (G) EDS elemental mapping images of FeS@NSC. The inset of (C) corresponds to the low-resolution SEM image of FeS@NSC.

To demonstrate the unique advantages of the strategy proposed herein, FeS@NSC nanowires were initially synthesized *via* our approach as a first demonstration. The high compositional purity of the FeS@NSC nanowires was clearly demonstrated by their X-ray diffraction (XRD) pattern (Fig. 1A) (JCPDS 65-6841). Furthermore, the Raman spectroscopy result (Fig. 1B) indicated the main characteristic peaks of the carbon materials, *i.e.*, the D-band at ∼1297 cm^−1^ and the G-band at ∼1583 cm^−1^, along with peaks assigned to the vibrational bands of Fe–S at ∼215 and ∼281 cm^−1^.^25^ More importantly, additional peaks at ∼390 and ∼475 cm^−1^ were also detected, which were assigned to the vibrational bands of the C–S–Fe and S–C bonds, respectively,^25^ further confirming the strong interactions between the carbon shell and the FeS core. The scanning electron microscopy (SEM) and transmission electron microscopy (TEM) images (Fig. 1C–E) revealed that the FeS@NSC nanowires were monodisperse and wrapped with a 8 nm-thick carbon shell layer. The *in situ* coated carbon layer would ideally alleviate the agglomeration problem caused by the high surface energy of the nano-FeS species during the synthetic process. In addition, the 1D nanowires increased the effective electrode–electrolyte contact area, enabling the electrolyte to access additional active sites on the electrode, and improved the utilization efficiency of the material.^26,27^ The high-resolution TEM image (HRTEM, Fig. 1F) illustrates the high crystallinity of the hexagonal FeS, with a lattice fringe of 0.21 nm corresponding to the (102) lattice plane. Additional energy dispersive X-ray spectroscopy (EDS) elemental mapping images (Fig. 1G) indicated a uniform distribution of carbon elements well wrapped around the single FeS nanowire, referring to a FeS content of 90.1 wt% by thermogravimetric analysis (TGA, Fig. S2†) and inductively coupled plasma-atomic emission spectroscopy. Moreover, nitrogen and sulfur elements were verified to be successfully doped in the carbon layer, suggesting plentiful heteroatoms doped in the outer carbon layer, which might be advantageous for the improvement of the electrochemical performance.

**Fig. 2:**
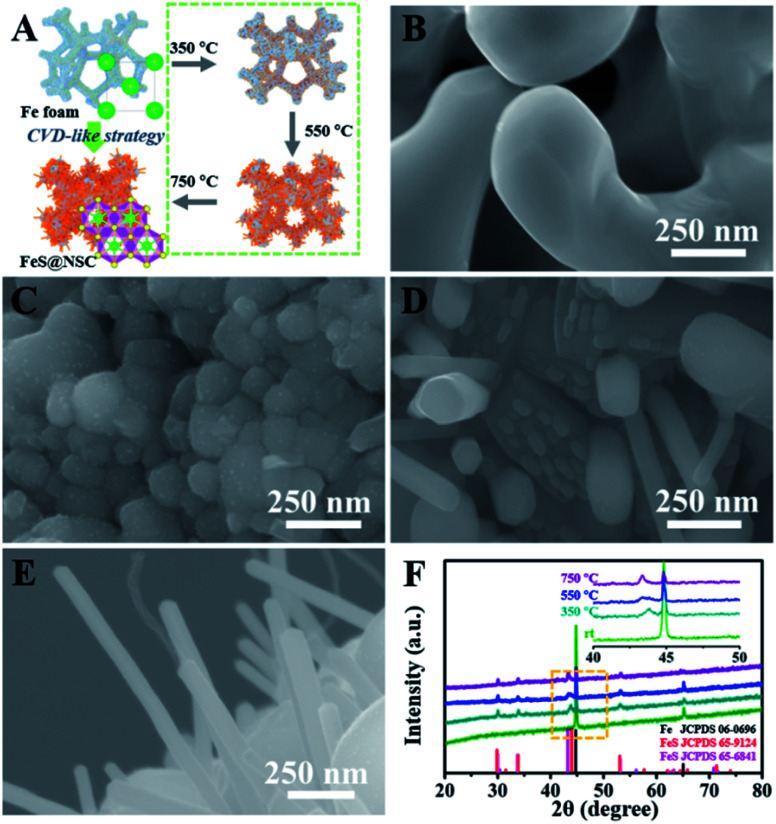
(A) Key stages of the growth of FeS@NSC by the CVD-like strategy. (B–E) High-resolution SEM images of Fe foam at (B) rt, (C) 350 °C, (D) 550 °C and (E) 750 °C. (F) Corresponding XRD patterns of Fe foam at each stage from rt to 750 °C. The inset of (F) shows the magnified XRD patterns at 40–50°.

To elucidate the growth of TMS@NSC heterostructured nanowires at the nanometer scale, the formation process of a typical FeS@NSC nanowire sample from Fe foam was further investigated. Fig. 2A outlines the key stages of the CVD-like growth process of the FeS@NSC nanowires at varying temperatures, corresponding to the structural evolution observations shown in Fig. 2B–E from room temperature (abbreviated as rt) to 750 °C. The formation process is defined as a “solid–gas–solid–liquid–solid” mechanism, where “solid” refers to the solid resin, “gas–solid–liquid” corresponds to the continuous process including decomposition of the resin to a sulfur-/carbon-containing gas, and the reaction of the gaseous precursor with metallic foam to form carbon-dissolved TMS solid solution, and the final “solid” means the generation of the TMS@NSC heterostructure after the dissolved carbon concentration reaches saturation. In detail, the magnified SEM image of the Fe foam (Fig. 2B) revealed a rough and porous surface with random Fe rod-like particles, which provided a larger surface area for the growth of FeS nanowires. The corresponding XRD pattern showed that the initial Fe foam (JCPDS 06-0696) contained a cubic crystal phase (Fig. 2F). The pyrolysis process of the resin was then investigated by TGA (Fig. S3A†), revealing an apparent mass loss at 90 °C, which was attributed to the loss of water from the resin (as further confirmed from the corresponding Fourier transform infrared spectroscopy (FTIR) results shown in Fig. S3B†). After heating to 245 °C, the resin further decomposed into CS_2_ and H_2_S, as indicated by the peaks at 1541 and 2353 cm^−1^, respectively, as well as NH_2_CN and other hydrocarbons,^28,29^ indicated by the peaks at 713, 2251, 3271, and 3338 cm^−1^. At 350 °C (Fig. 2C and F), the generated CS_2_ and H_2_S reacted with the Fe foam, resulting in the formation of spherical hexagonal FeS (*P*6_3_/*mmc*) particles on the surface of iron foam. Simultaneously, carbon atoms originating from the generated hydrocarbons dissolved in the formed FeS and formed the outer carbon layer once the carbon concentration was saturated, leading to the core–shell FeS@NSC heterostructure. At 550 °C (Fig. 2D and F), each spherical particle further grew to form a rod prototype and some short nanorods, with the lattice plane (102) of FeS shifting to a lower angle closer to the standard peak, indicating that more perfect crystalline FeS was obtained. When further increasing to 750 °C (Fig. 2E and F), hexagonal FeS@NSC nanorods (JCPDS 65-6841) further grew into long nanowires with diameters of 70–120 nm.

The versatility of our CVD-like strategy was further demonstrated by growing TMS@NSC heterostructured nanowires on other metallic foams, including Co and Cu foams. The XRD, SEM, TEM, and HRTEM, as well as the EDS elemental mapping images of the representative samples, further confirmed the successful formation of other 1D core–shell heterostructures, including Co_9_S_8_@NSC and Cu_2_S@NSC (Fig. S4 and S5†). These results implied that the formation of 1D core–shell TMS@NSC heterostructured nanowires on metallic foams is, in general, independent of the composition and species of the metallic substrates.

To highlight the advantageous features of the FeS@NSC nanowires, other FeS-based nanomaterials were also prepared for comparison. The XRD patterns of commercial FeS, FeS/NSC, and NSC samples (Fig. S6†) revealed four dominant peaks located at 29.9°, 33.7°, 43.2°, and 53.1°, corresponding to the (100), (101), (102), and (110) lattice planes of the hexagonal structured FeS, respectively (JCPDS 65-6841). Additionally, irregularly shaped small particles were observed in the SEM images of the commercial FeS materials (Fig. S7A and B†). The corresponding EDS elemental mapping images indicated the good overlapping of the Fe and S particle distributions (Fig. S7C and D†). Furthermore, the TEM and HRTEM images of the commercial FeS sample (Fig. S7E and F†) revealed the interplanar distances of 0.21 and 0.26 nm, associated with the (102) and (101) lattice planes, respectively. The NSC exhibited an irregular morphology with particle sizes in the range of 1–5 μm and random distribution of N and S elements (Fig. S8†), indicating the presence of N and S dopants in the obtained matrix. The obtained FeS/NSC bulk exhibited an irregular morphology with poor and random contacts between the NSC and FeS particles (Fig. S9†), revealing that the direct pyrolysis of the precursors could not achieve 1D core–shell heterostructures.

**Fig. 3:**
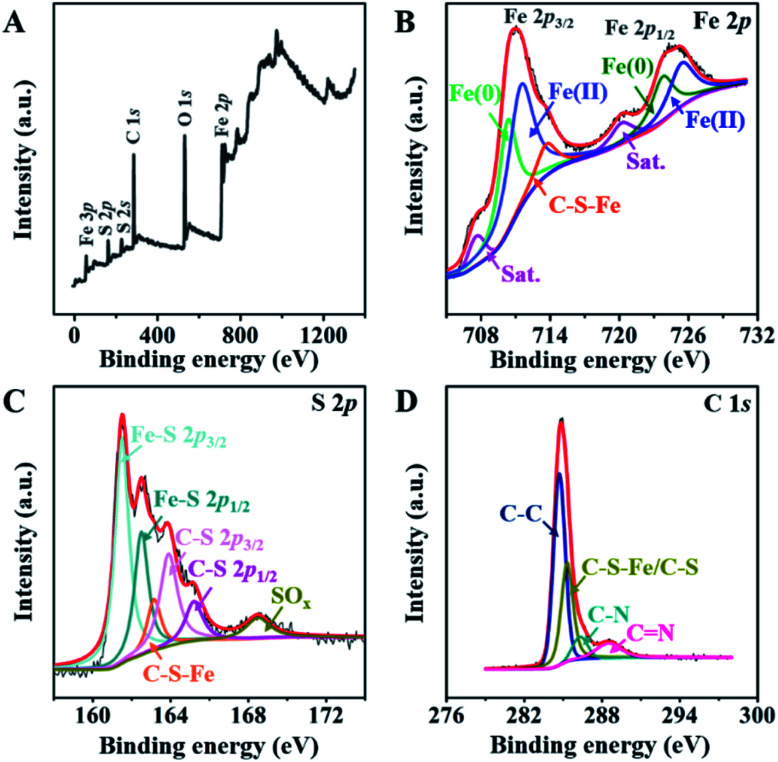
(A)The full XPS spectrum, and high resolution XPS spectra of (B) Fe 2p, (C) S 2p, and (D) C 1s of FeS@NSC.

The chemical compositions of the FeS@NSC nanowires and FeS were initially studied by FTIR spectra in the range of 250–2000 cm^−1^ (Fig. S10†), revealing an obscure band at 1100 cm^−1^ (attributed to the vibrations of Fe–S–C/S–C), two bands at 886 and 607 cm^−1^ (corresponding to the vibrations of S–O and Fe–S, respectively),^30^ and one band at 1620 cm^−1^ (attributed to the vibration of –C=O).^31^ The X-ray photoelectron spectroscopy (XPS) spectrum of the FeS@NSC nanowires (Fig. 3A) further revealed the details of their chemical compositions. The Fe 2p spectrum (Fig. 3B) exhibited a significant peak which was assigned to the C–S–Fe bonds between FeS and the carbon shell,^32,33^ indicating the strong FeS–carbon shell interaction generated *via* our CVD-like strategy. In addition, other peaks attributed to Fe 2p_3/2_ and Fe 2p_1/2_ (ref. 34 and 35) were observed. The S 2p spectrum exhibited an obvious peak assignable to the C–S–Fe bonds (Fig. 3C), which was consistent with the Fe 2p spectrum (Fig. 3B). Besides, four additional peaks were attributed to Fe–S and C–S bonds, and the peak assigned to the oxidation groups formed by S^2−^ in air^36,37^ was also observed at a higher binding energy. In the C 1s spectrum (Fig. 3D), the four peaks were characteristic of the C–C, C–S–Fe/C–S, C–N, and C=N bonds, respectively.^38,39^ The successful doping of nitrogen and sulfur atoms into the carbon matrix not only improved the conductivity of the material, but also catalyzed the oxidation of polysulfides in sodium-ion batteries, thereby inhibiting their dissolution and decelerating the capacity fading.^40,41^

**Fig. 4:**
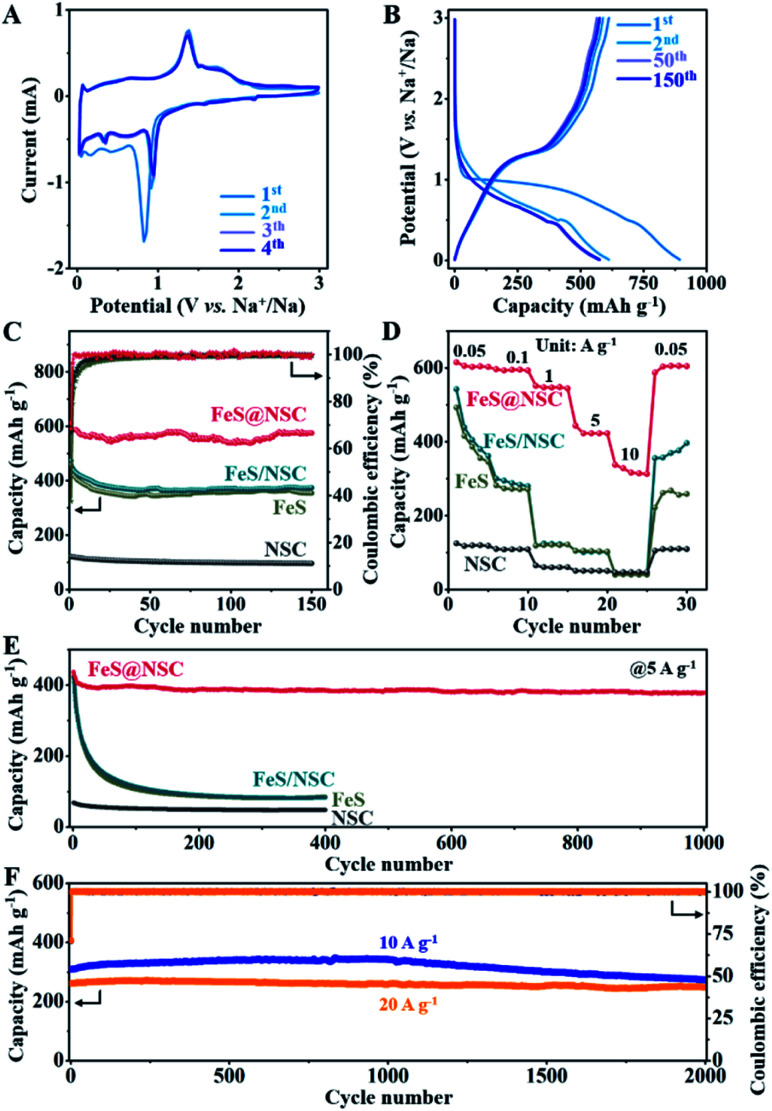
(A) CV curves of FeS@NSC; (B) charge/discharge curves of FeS@NSC at 0.1 A g^−1^; (C) charge capacities and corresponding coulombic efficiencies at 0.1 A g^−1^; (D) rate capacities for current densities in the range of 0.05–10 A g^−1^ between 0.01 and 3 V (*vs.* Na^+^/Na); (E) charge capacities of FeS@NSC, FeS, FeS/NSC, and NSC composites at 5 A g^−1^; (F) charge capacities of the FeS@NSC composite at 10 and 20 A g^−1^.

To comparatively evaluate the sodium-ion storage performance of our fabricated FeS@NSC nanowires, FeS, NSC, and FeS/NSC were also investigated using a half-cell battery with a Na-based counter electrode. Fig. 4A and S11–S13† present the cyclic voltammetry (CV) curves of the samples in a voltage window of 0.01–3 V (*vs.* Na^+^/Na) at 0.1 mV s^−1^. For the FeS@NSC nanowires (Fig. 4A), the cathodic peaks at ∼0.82, ∼0.41, and ∼0.17 V of the 1st cathodic curve were assigned to the electrochemical redox reaction of FeS and Na^+^ to form Fe and Na_2_S, and influence of the solid electrolyte interphase (SEI) film.^36^ The two peaks at 1.37 and ∼1.74 V of the first anodic curve were attributed to the transition of Fe and Na_2_S to Na_*x*_FeS and FeS during the desodiation process,^34^ which then appeared at ∼0.92 and ∼0.35 V, respectively, in the following three cathodic curves, corresponding to the sodiation process. Moreover, a pair of reduction/oxidation peaks which appeared at ∼0.03 and 0.06 V were attributed to the Na^+^ insertion/extraction processes in the carbon matrix, respectively,^42^ a behavior which was consistent with the results of the NSC sample (Fig. S12†). The 2nd to 4th CV cycles overlapped well, indicating the excellent cyclability of FeS@NSC. In contrast, the reduction peaks of FeS and FeS/NSC at 0.92 V were not visible. This was ascribed to the poor kinetics of the reaction of FeS and FeS/NSC with Na^+^, resulting in an incomplete reaction with sodium ions and a considerably lower specific capacity than that of FeS@NSC. Fig. 4B illustrates a comparison of the 1st, 2nd, 50th, and 150th cycles of the galvanostatic charge/discharge profiles of the FeS@NSC electrode at a current density of 100 mA g^−1^. The obtained constant-current charge/discharge platform was consistent with the oxidation/reduction peak of the CV curve.

Fig. 4C demonstrates that the first charge/discharge capacity of the FeS@NSC electrode was 892.8/611.6 mA h g^−1^, and the initial coulombic efficiency was 68.5%. After 150 cycles, the FeS@NSC sample retained a specific capacity of 575.0 mA h g^−1^, corresponding to a capacity attenuation rate of 94.0%. The reversible specific capacities of the FeS/NSC, FeS, and NSC samples in the 150th cycle were 373.9, 354.1, and 95.8 mA h g^−1^, respectively. This downgrade was ascribed to a combination of various factors, such as reactions with surface functional groups, formation and stabilization of the SEI film, and the irreversible intercalation of Na^+^. As the current density increased from 0.05 to 0.1, 1, 5, and 10 A g^−1^, the FeS@NSC electrode delivered decreasing capacities of 615.1, 596.5, 551.5, 443.7, and 337.7 mA h g^−1^, respectively (Fig. 4D). More importantly, when the original current density of 0.05 A g^−1^ was again reintroduced, the FeS@NSC electrode rapidly attained a capacity of 601.3 mA h g^−1^, indicating that 97.8% of the capacity was effectively recovered. However, at the same current density, the specific capacities of the FeS/NSC, FeS, and NSC electrodes were significantly lower than that of FeS@NSC. These results further verified that the rate capability of FeS could be effectively improved *via in situ* coating with a nitrogen-/sulfur-double-doped carbon layer.

As illustrated in Fig. 4E, the FeS@NSC electrode exhibited a reversible capacity of 378.6 mA h g^−1^ after 1000 charge/discharge cycles at 5 A g^−1^. In contrast, the FeS/NSC, FeS, and NSC electrodes exhibited notably lower capacities after 400 cycles (85.7, 82.6, and 47.6 mA h g^−1^, respectively). The FeS@NSC anode delivered the reversible specific capacities of 309.3 and 261.7 mA h g^−1^ at the rates of 10 and 20 A g^−1^ (Fig. 4F), respectively. After 2000 cycles, the FeS@NSC electrode successfully retained the specific capacities of 273.2 and 247.8 mA h g^−1^ at 10 and 20 A g^−1^, respectively, corresponding to the capacity retention rates of 88.3% and 94.7% and capacity attenuation rates of 0.0058 and 0.0027% per cycle, respectively. Simultaneously, compared with recently reported iron sulfide-based materials and other metal sulfides, the FeS@NSC nanowires not only had an advantageous synthesis procedure, but also exhibited both superior reversible capacity and higher cycling stability (Table S1†). This was due to the fact that the prepared 1D FeS nanowires with *in situ* formed N,S double-doped carbon layer provided a stable one-way electron transport path, consequently reducing the charge transport resistance, improving the conductivity and dynamics of the electrode, and achieving a high current charge/discharge performance.^43^ This phenomenon was also evidenced by the typical Nyquist curves obtained by the electrochemical impedance spectroscopy (EIS) analysis of the four samples (Fig. S14†).

**Fig. 5:**
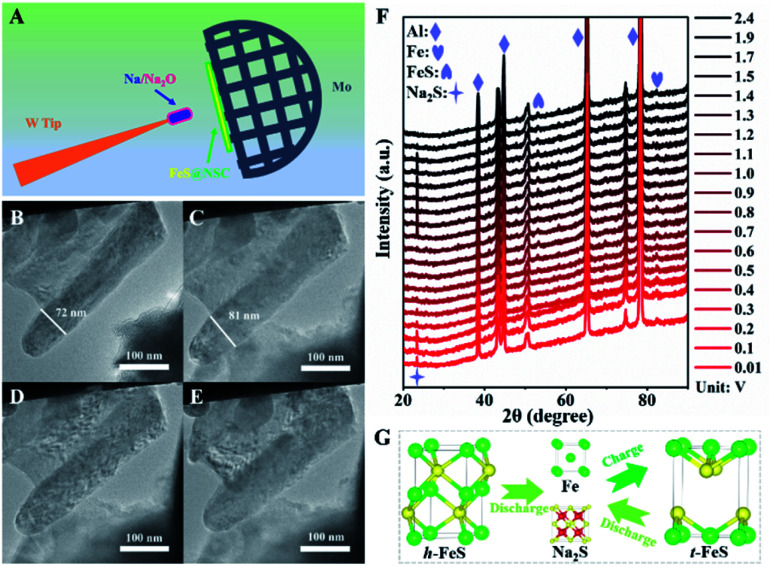
(A) Schematic illustration of *in situ* TEM experiment during the sodiation process; (B–E) TEM images of FeS@NSC during different sodiation/desodiation processes: (B) initial stage, (C) sodiation, (D) desodiation, and (E) sodiation; (F) *in situ* XRD patterns of the FeS@NSC electrode after the initial discharge; (G) atomic model of the phase change of FeS@NSC during the discharge and charge processes.

Next, we explored the microstructural and morphological evolution of the FeS@NSC composite during the sodiation/desodiation processes using *in situ* TEM and *in situ* XRD (Fig. 5). The FeS@NSC electrode and metallic Na/Na_2_O were fixed on the Mo and W tips to act as the working and counter electrodes, respectively, as shown in Fig. 5A. The corresponding morphological evoluations of a single FeS@NSC nanowire confirmed through *in situ* TEM observations could be seen from ESI movie 1–3.†Fig. 5B shows the TEM image of a single FeS@NSC nanowire before sodiation; the selected area electron diffraction (SAED; Fig. S15A†) image shows clear diffraction rings for the (101), (102), and (202) planes of hexagonal FeS (denoted as *h*-FeS). During the sodiation process, the diameter of the nanowires increased from 72 to 81 nm (Fig. 5C), corresponding to a low volume expansion rate of ∼12.5%, revealing that the generated carbon shell significantly inhibited the volume expansion of FeS during the sodiation process. The corresponding electron diffraction analysis result (Fig. S15B†) shows rings of Fe and Na_2_S with a cubic crystal structure. Intriguingly, instead of the original *h*-FeS phase, a new crystalline phase of tetragonal FeS (*t*-FeS) was formed after the desodiation stage in the 1st cycle, as shown in Fig. 5D and S15C.† In the subsequent charge/discharge cycle, the *t*-FeS phase was converted into “Fe + Na_2_S” (Fig. 5E and S15D†), and the presence of *t*-FeS was confirmed by TEM analysis after 150 cycles (Fig. S16†), indicating the high reversibility of *t*-FeS during the sodiation/desodiation cycles which favors long-term cycling. The overall electrochemical process has also been confirmed by *in situ* XRD observations (Fig. 5F), demonstrating the phase transformation from *h*-FeS to “Fe + Na_2_S” (Fig. 5G). Notably, compared with commercial Fe, which exhibited numerous crushed and aggregated small particles after cycling (Fig. S17†), FeS@NSC retained its structural pattern of core–shell nanowires after 150 sodiation/desodiation cycles, indicating its excellent structural stability during extreme cycling processing. The excellent capacity, superior rate performance, and ultrahigh cycling stability of FeS@NSC could be attributed to its unique architecture and phase transition, as FeS@NSC provided: (i) a highly stable structure, owing to the strong interaction of C–S–Fe bonds between the carbon shell and FeS; (ii) fast Na^+^ diffusion kinetics enabled by the 1D co-axial FeS@NSC nanowires; (iii) a large contact area between FeS@NSC and the electrolyte; (iv) fast electron transport and buffering of volume variations, originating from the uniform N,S double-doped carbon shell; and (v) the crystalline phase transition of the *h*-FeS nanowires during the 1st cycle and the high reversibility of *t*-FeS in the subsequent cycles.

## Conclusions

In summary, we developed a universal strategy to synthesize 1D core–shell TMS@NSC heterostructured nanowires by employing a CVD-like technique and using a solid phase resin and metallic foam as the precursors. The growth and sodiation/desodiation processes of FeS@NSC were characterized in detail. Intriguingly, it was revealed for the first time that FeS nanowires in FeS@NSC underwent a crystalline phase transition from *h*-FeS to highly reversible *t*-FeS during the 1st charge/discharge cycle, as detected by *in situ* TEM and *in situ* XRD. In addition to the crystalline phase transition, the unique and advantageous nanostructure and *in situ*-generated C–S–Fe bonds between FeS and the carbon shell significantly benefitted the electrochemical performance of FeS. As a result, FeS@NSC delivered a high reversible capacity of 611.6 mA h g^−1^ and retained a capacity of 575.0 mA h g^−1^ after 150 charge/discharge cycles at 100 mA g^−1^. At a current density of 20 A g^−1^, FeS@NSC exhibited a reversible specific capacity of 261.7 mA h g^−1^ and a capacity of 247.8 mA h g^−1^ after 2000 charge/discharge cycles, corresponding to a capacity decay rate of 0.0027% per cycle and a capacity retention rate of 94.7%. Stemming from its universal, facile, cost-effective, and efficient features, our proposed strategy paves the way for the effective synthesis of core–shell carbon *in situ*-coated metal sulfides for advanced energy storage applications.

## Data availability

The data that support the findings of this study are available within the article and its ESI,† or from the corresponding authors on reasonable request.

## Author contributions

J. Zhu, X. Xu and Y. Yamauchi: supervision and funding acquisition. They designed the project, designed the experiments and edited the manuscript. P. Wei, J. Zhu and Y. Qiu performed the synthesis, characterization and electrochemical measurements, as well as wrote the first draft of the manuscript. G. Wang, S. Ma, P. K. Shen and X.-L. Wu provided critical advice during the manuscript writing.

## Conflicts of interest

There are no conflicts to declare.

## Supplementary Material

SC-012-D1SC04163K-s001

SC-012-D1SC04163K-s002

SC-012-D1SC04163K-s003

SC-012-D1SC04163K-s004
