# Supplementary material for: One-dimensional core–shell motif nanowires with chemically-bonded transition metal sulfide-carbon heterostructures for efficient sodium-ion storage
Source: Chem Sci. 2021 Oct 27;12(45):15054–60. doi: 10.1039/d1sc04163k (PMC8612380; doi:10.1039/d1sc04163k)
Supplement: SC-012-D1SC04163K-s001 [file SC-012-D1SC04163K-s001.pdf]

Electronic Supplementary Material (ESI) for Chemical Science.
This journal is © The Royal Society of Chemistry 2021

Experimental Section

Synthesis of Co_9S_8@NSC: First, 0.5 g of nitrogen and sulfur-containing resin (Suqing Group, China) was added to the square corundum, and then covered by cobalt foam ($3 \times 6 \times 0.15$ cm^3). Then, the temperature was increased to 700 °C under a nitrogen atmosphere, where it was preserved for 2 h.

Preparation of Cu_2S@NSC: The synthetic process was performed by following the exact same procedure as that described above, with the following exceptions: the copper foam was replaced by cobalt foam and the heating temperature was changed to 1000 °C.

Preparation of FeS@NSC: The cobalt foam was replaced by iron foam and the constant heating temperature was changed to 750 °C; otherwise, the conditions were the same as those for the synthesis of Co_9S_8@NSC nanorods. For comparison, nitrogen and sulfur double-doped carbon (NSC) was prepared by heat treatment of nitrogen and sulfur-containing resin for 2 h in the absence of metal foam. A mixture of FeS and NSC (FeS/NSC) was prepared by mechanically mixing commercial FeS with NSC in a mass ratio of 9:1.

Physical Characterization

The structural and morphological characteristics, along with the phase content and elemental composition of the composite samples, were analyzed by transmission electron microscopy (TEM, Titan ETEM G2 80–300, FEI Company), scanning electron microscopy (SEM, FESEM SU8220, Hitachi), X-ray diffraction (XRD, SmartLab, Rigaku Corporation; Cu Kα radiation), and X-ray photoelectron spectroscopy (XPS, ESCALAB 250, Thermo-VG Scientific). The FeS content was characterized by inductively coupled plasma-atomic emission spectrometry (ICP) and a differential scanning calorimeter/thermogravimetric analyzer (DSC/TGA, STA449-F5 TAQ600) in an air atmosphere from 25 to 800 °C.

Electrochemical characterization

Since the adhesion interaction between TMS@NSC and the metallic foam is strong, it is easy to obtain TMS@NSC through clamping iron foam between two metal titanium sheets with a further squeezing operation on the oil press. After that, the FeS@NSC, polyvinylidene fluoride (PVDF), and acetylene black were mixed in a weight ratio of 8:1:1 and added to a certain amount of N-methyl-2-pyrrolidone (NMP). After stirring for 4 h, the collected slurry was uniformly coated on a Cu foil and left to dry in a vacuum oven at 100 °C overnight. The resulting electrode was cut into pieces to obtain 1.4 cm-diameter discs. The average mass loading of the

electrode was about 1.0 mg cm^{-2}. Porous glass fiber membranes were employed as separators, and 1 M $NaPF_6$ dissolved in dimethyl carbonate (DMC) served as the electrolyte. The cyclic performance and rate capability were determined using a battery testing system (Shenzhen Neware Battery, China) with galvanostatic charge-discharge measurements performed between 0.01 and 3 V (*vs.* Na^+/Na). Cyclic voltammetry (CV; scan rate: 0.1 mV s^{-1}) and electrochemical impedance spectroscopy (EIS) analyses were carried out on an electrochemical workstation (IM6, Zahner-Elektrik, Germany) at frequencies from 100 kHz to 10 mHz with a disturbance amplitude of 5 mV.

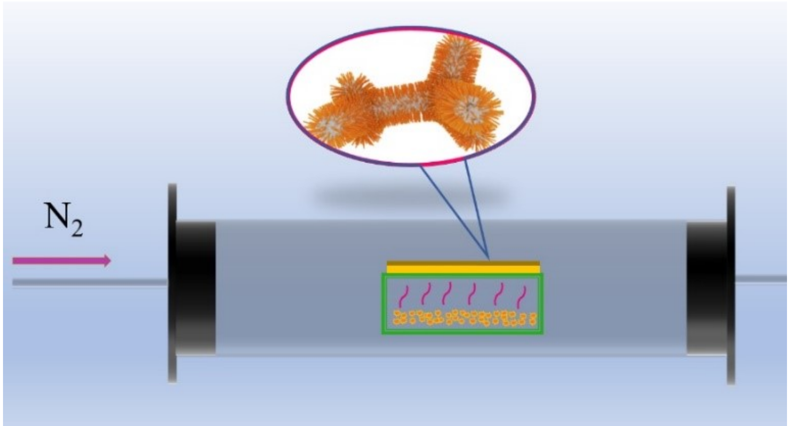

Fig. S1 Schematic illustration of the general synthetic process for the TMS@NSC nanowires.

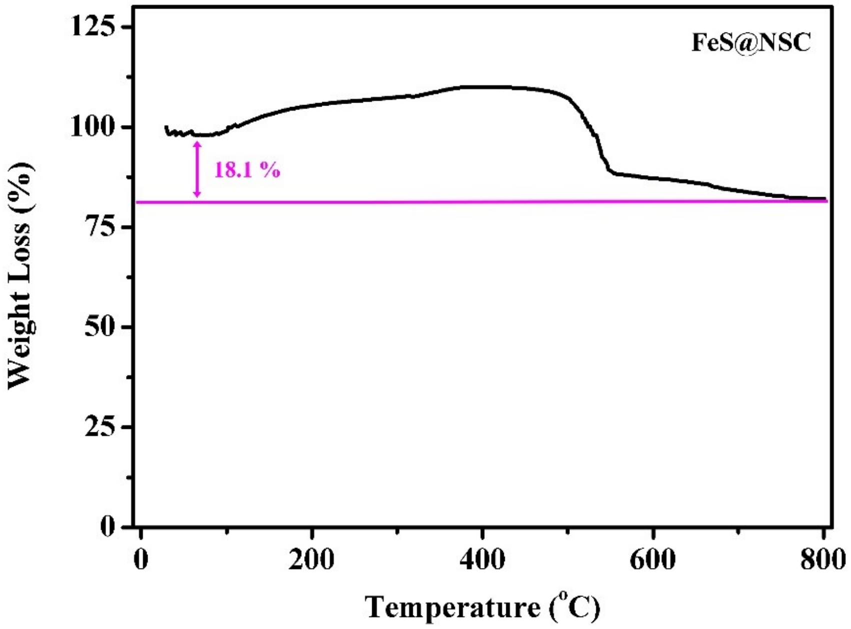

Fig. S2 TGA curve of the FeS@NSC tested in air.

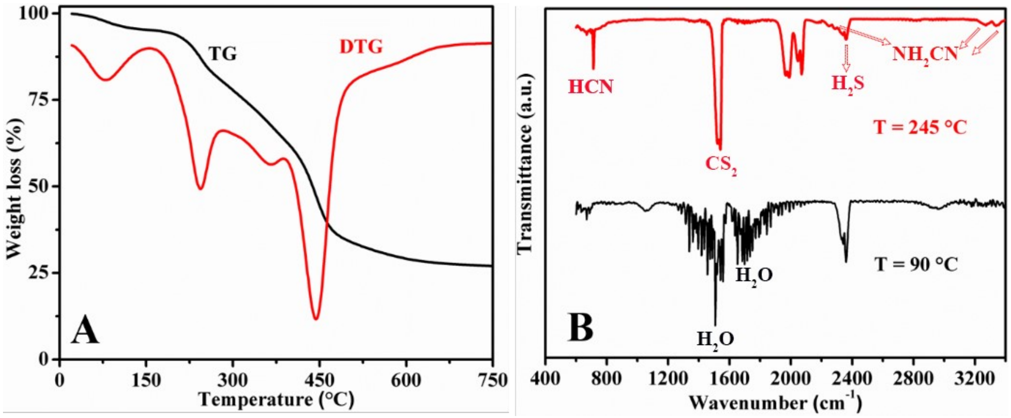

Fig. S3 (A) TG-DTG results for the resin in N_2 and (B) FTIR spectra of the corresponding pyrolysis products at 90 and 245 °C.

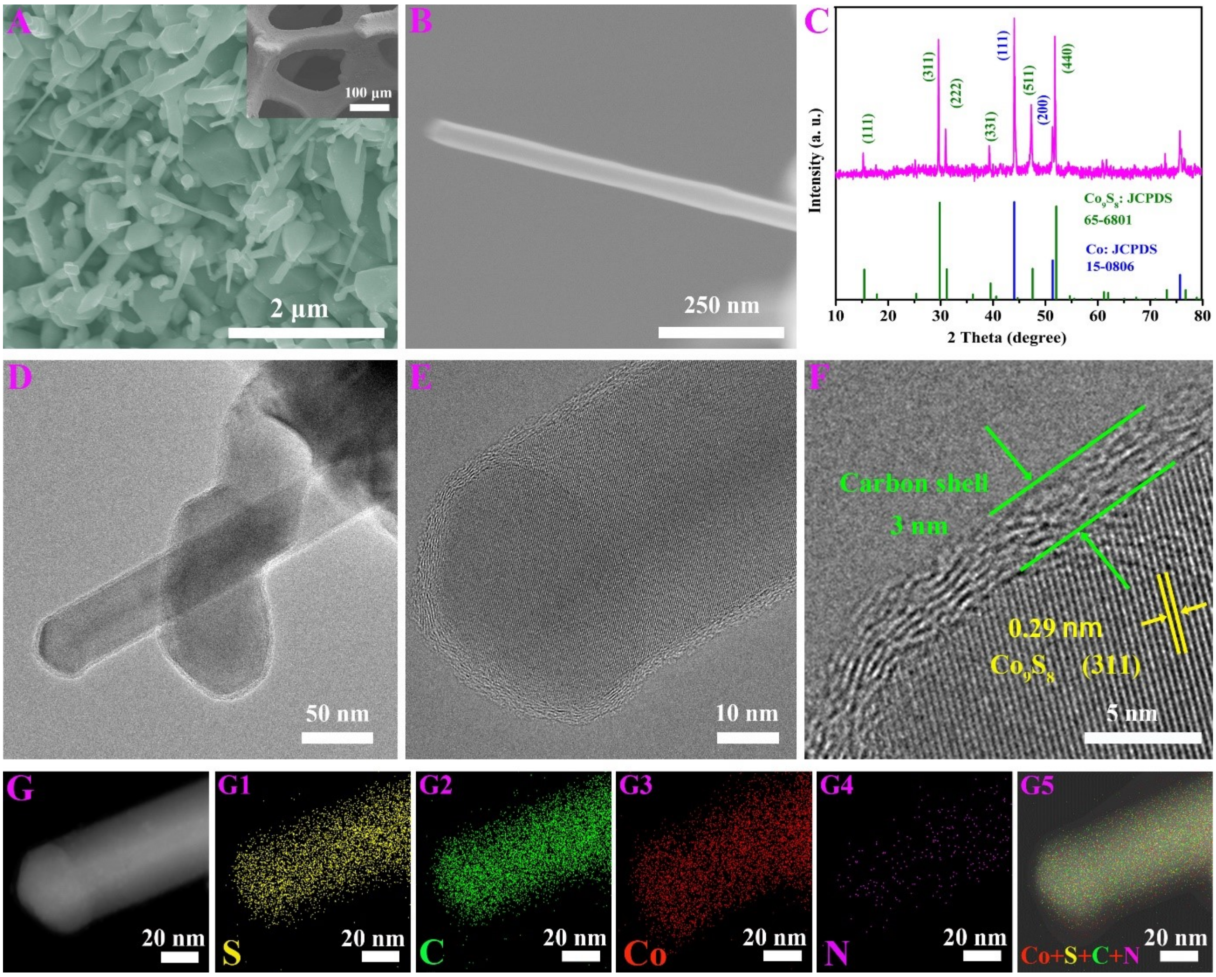

Fig. S4 (A, B) SEM images, (C) XRD, and (D-F) TEM and HRTEM images of Co_9S_8@NSC; (G) dark-field TEM and EDS elemental mapping images of Co_9S_8@NSC.

Supplementary Note for Fig. S4: Fig. S4A, B illustrated that the entire Co foam surface was thickly covered with nanorods, with each nanorod being approximately 50 nm in diameter and 1 µm in length. The XRD pattern (**Fig. S4C**) showed that the produced Co_9S_8 (JCPDS 65-6801) had a cubic crystal structure with the space group (SG) *Fm3m* (225). In good agreement with the SEM results, TEM analysis showed that the diameter of the nanorods was 50 nm. The HRTEM images (**Fig. S4D-F**) showed that Co_9S_8 was wrapped by a uniform 3 nm-thick carbon shell and its large interplanar crystal (length=0.29 nm) was characterized by (311) crystal planes. The dark-field TEM and EDS elemental mapping images of Co_9S_8@NSC showed that the carbon species were uniformly distributed (**Fig. S4G**), and the carbon layer was well coated on the outer layer of Co_9S_8. The distributions of S and Co atoms overlapped very well in the core, and a very small amount of S was sparsely and randomly distributed on the edges, indicating that a small amount of S was doped in the carbon layer.

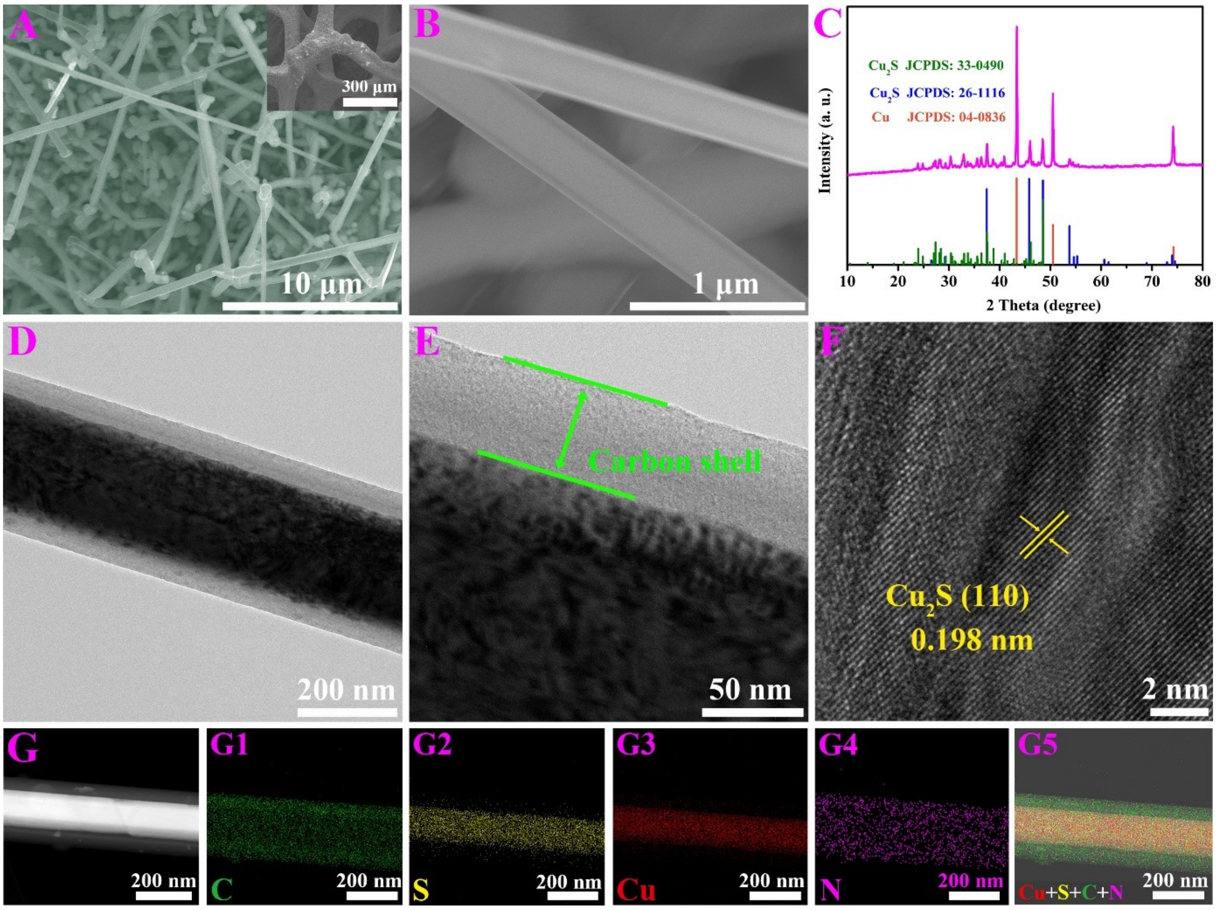

Fig. S5 (A, B) SEM images, (C) XRD, and (D-F) TEM and HRTEM images of Cu_2S@NSC; (G) dark-field TEM and EDS elemental mapping images of Cu_2S@NSC.

Supplementary Note for Fig. S5: Fig. S5A, B showed the SEM image of Cu_2S@NSC grown on the surface of copper foam with Cu_2S encapsulated in a carbon shell. The XRD pattern of the formed Cu_2S revealed two crystal structures, *i.e.*, monoclinic (JCPDS 33-0490) and hexagonal (JCPDS 26-1116) crystal structures (**Fig. S5C**). The HRTEM images (**Fig. S5D-F**) revealed that Cu_2S with a large interplanar crystal length of 0.198 nm was associated with the (110) crystal plane. The thickness of the carbon shell was approximately 50 nm. The dark-field TEM image and EDS elemental mapping images of Cu_2S@NSC showed that the distribution areas of C, S, and N possessed the same widths but exhibited obvious differences in their respective element distribution density (**Fig. S5G**). It is worth noting that the distribution of Cu was narrower than those of C, S, and N. The distribution of S not only overlapped with that of Cu in the core area of the submicron rods, but it was also randomly and sparsely scattered outside the nanorod core area. In contrast, C demonstrated a higher distribution density on the outside of the nanorods than in the central area.

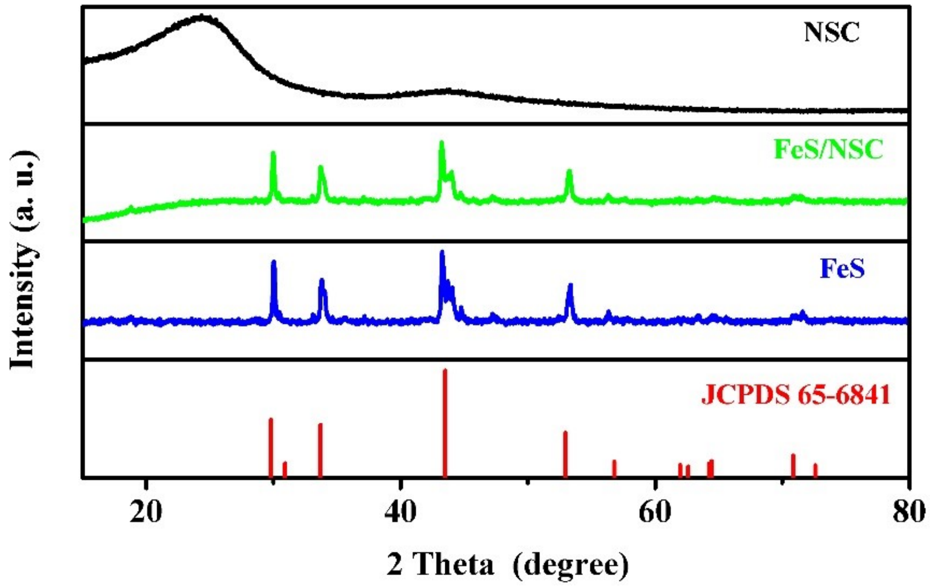

Fig. S6 XRD patterns of FeS, FeS/NSC and NSC.

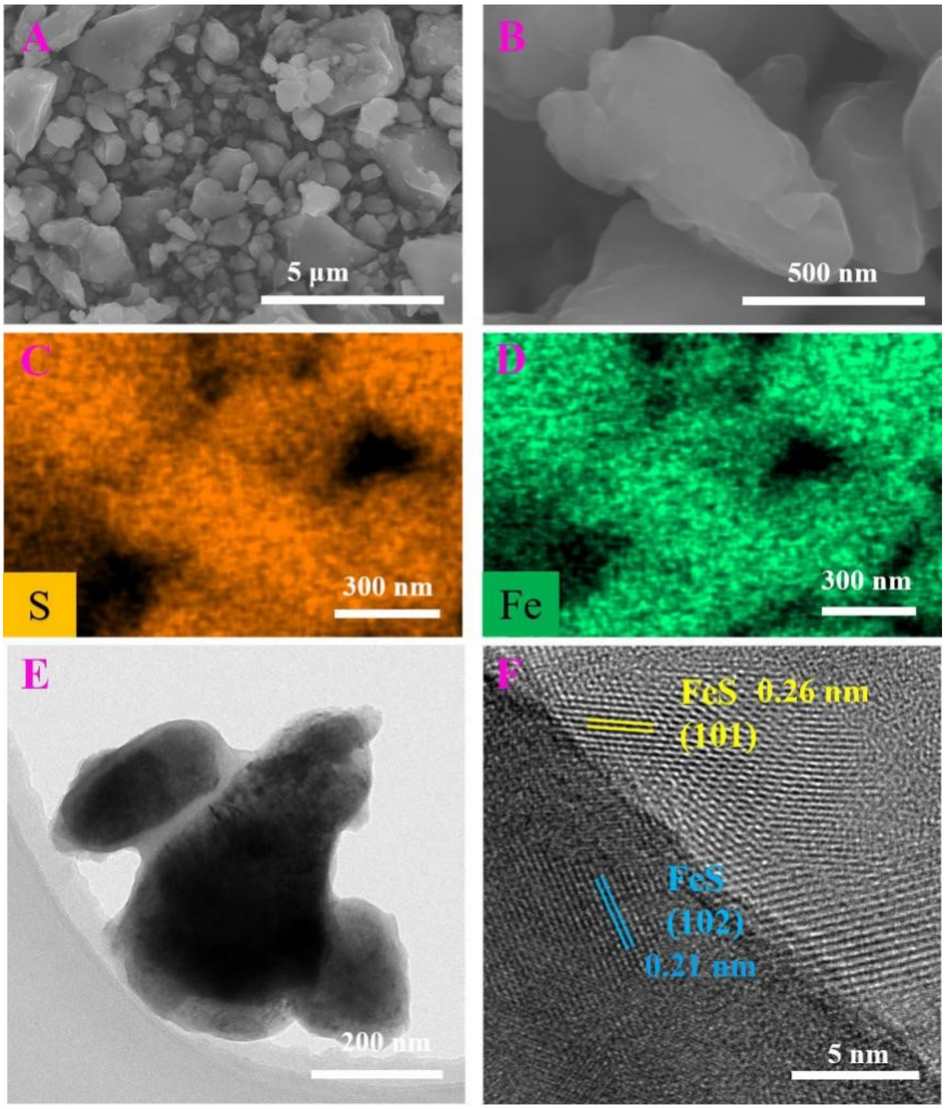

Fig. S7 (A, B) SEM, (C, D) EDS elemental mapping, (E) TEM and (F) HRTEM images of FeS.

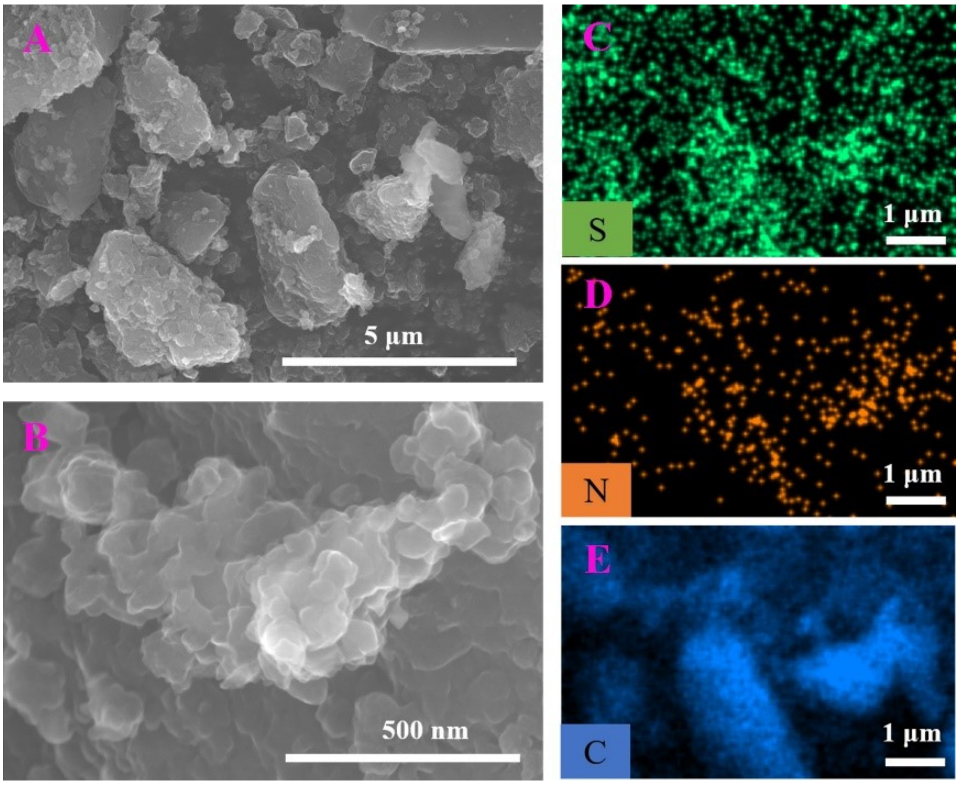

Fig. S8 (A, B) SEM and (C-E) EDS elemental mapping images of NSC.

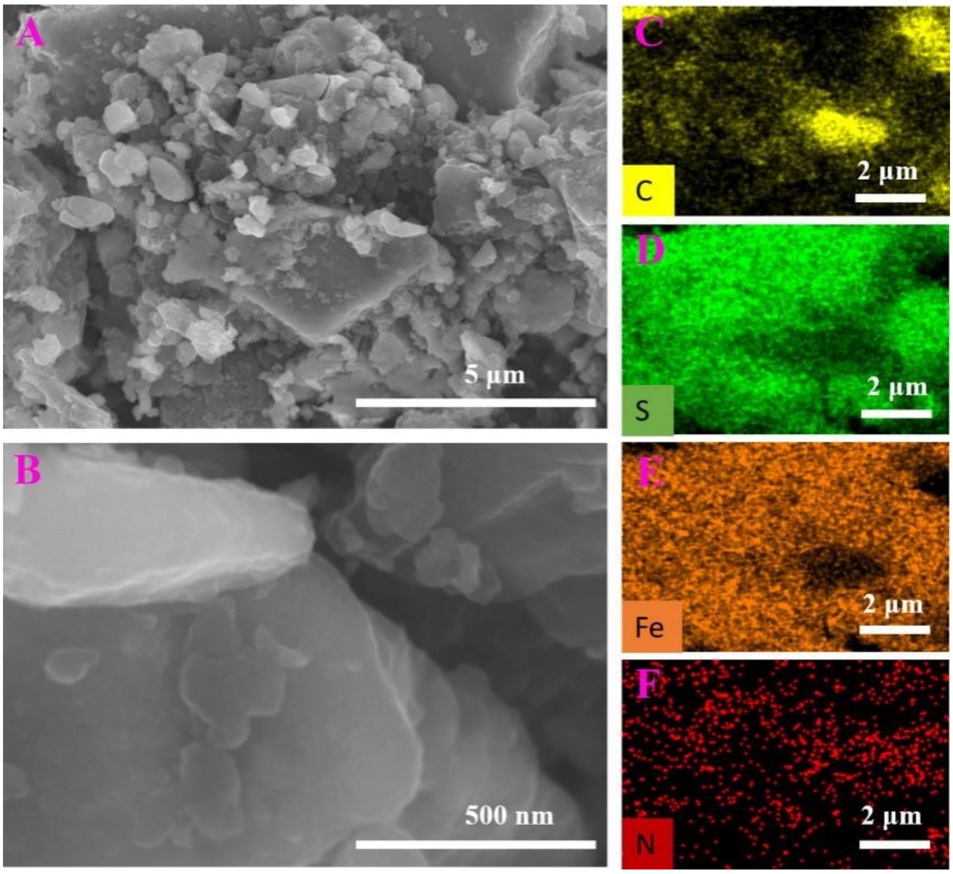

Fig. S9 (A, B) SEM and (C-F) EDS elemental mapping images of FeS/NSC.

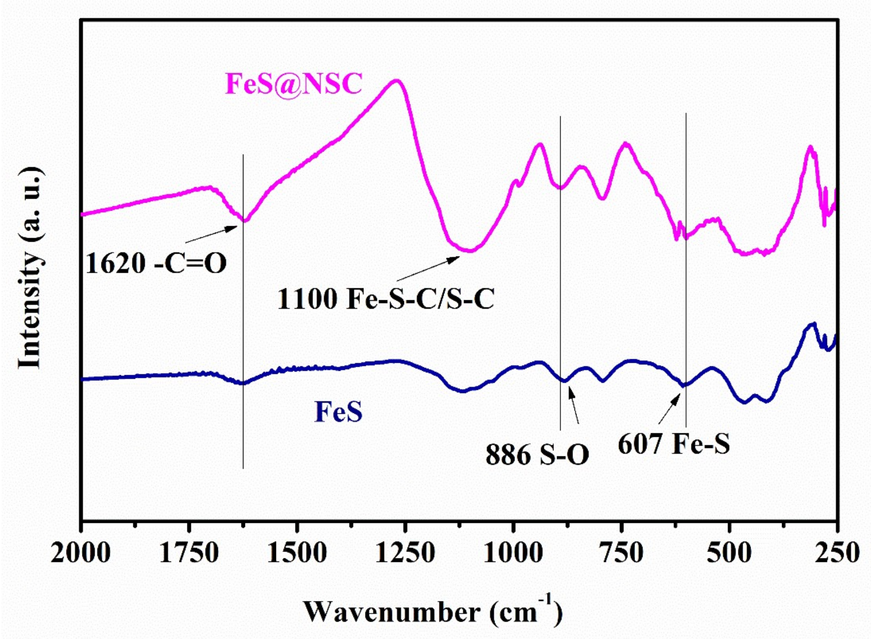

Fig. S10 FTIR spectra of FeS@NSC and FeS.

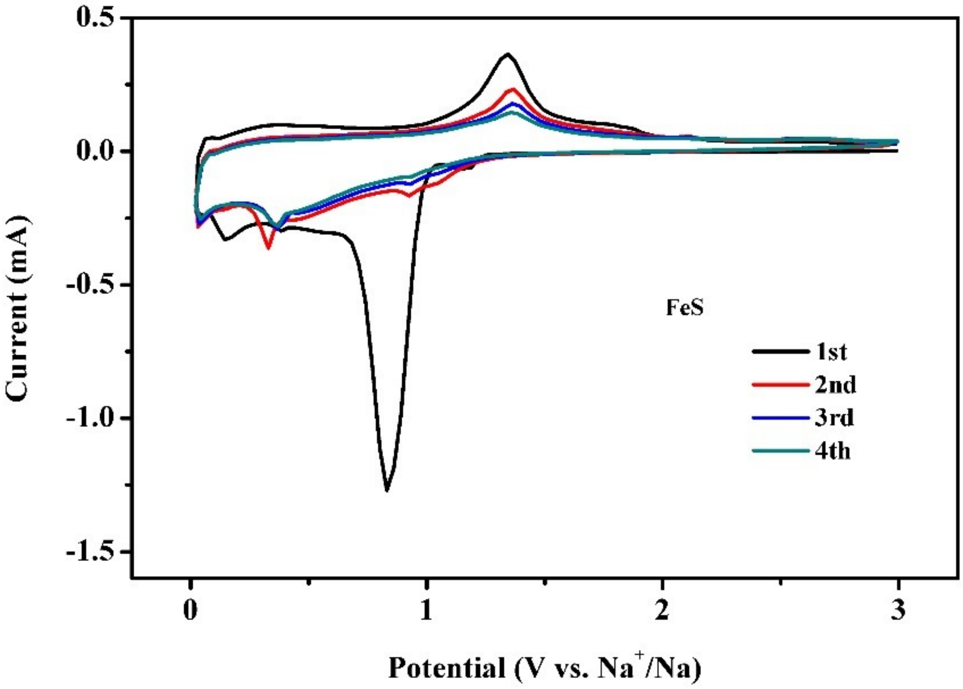

Fig. S11 CV curves of FeS.

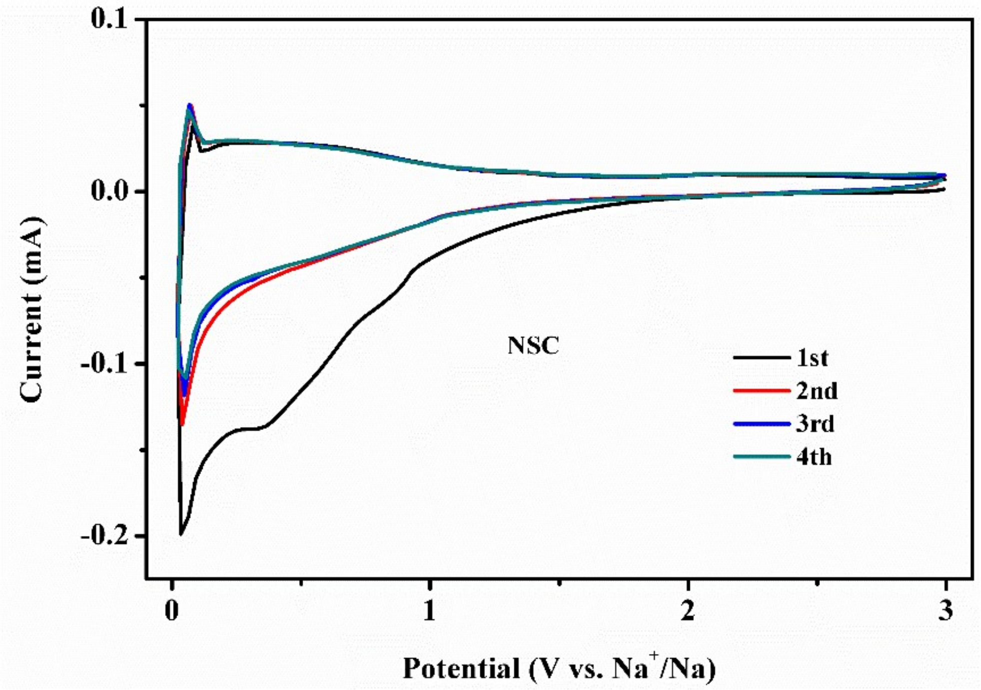

Fig. S12 CV curves of NSC.

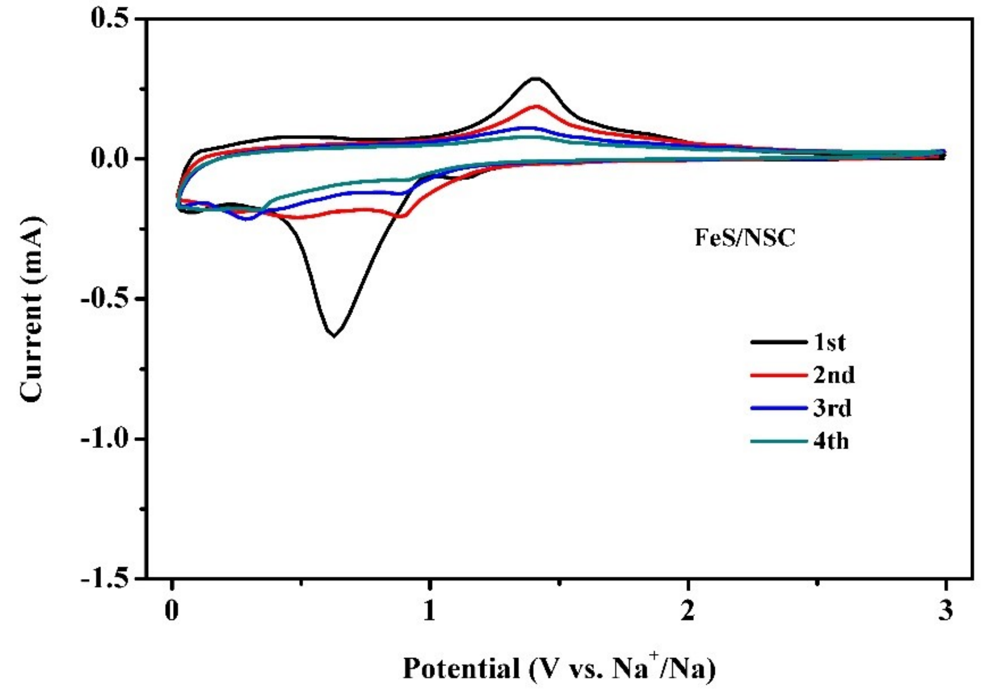

Fig. S13 CV curves of FeS/NSC.

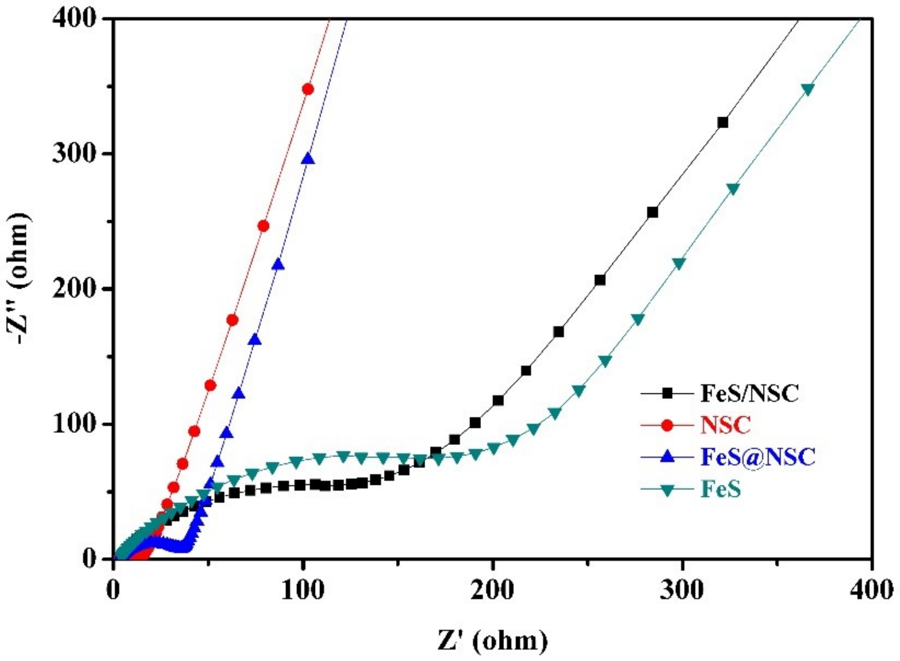

Fig. S14 Nyquist plots of FeS@NSC, FeS/NSC, FeS and NSC.

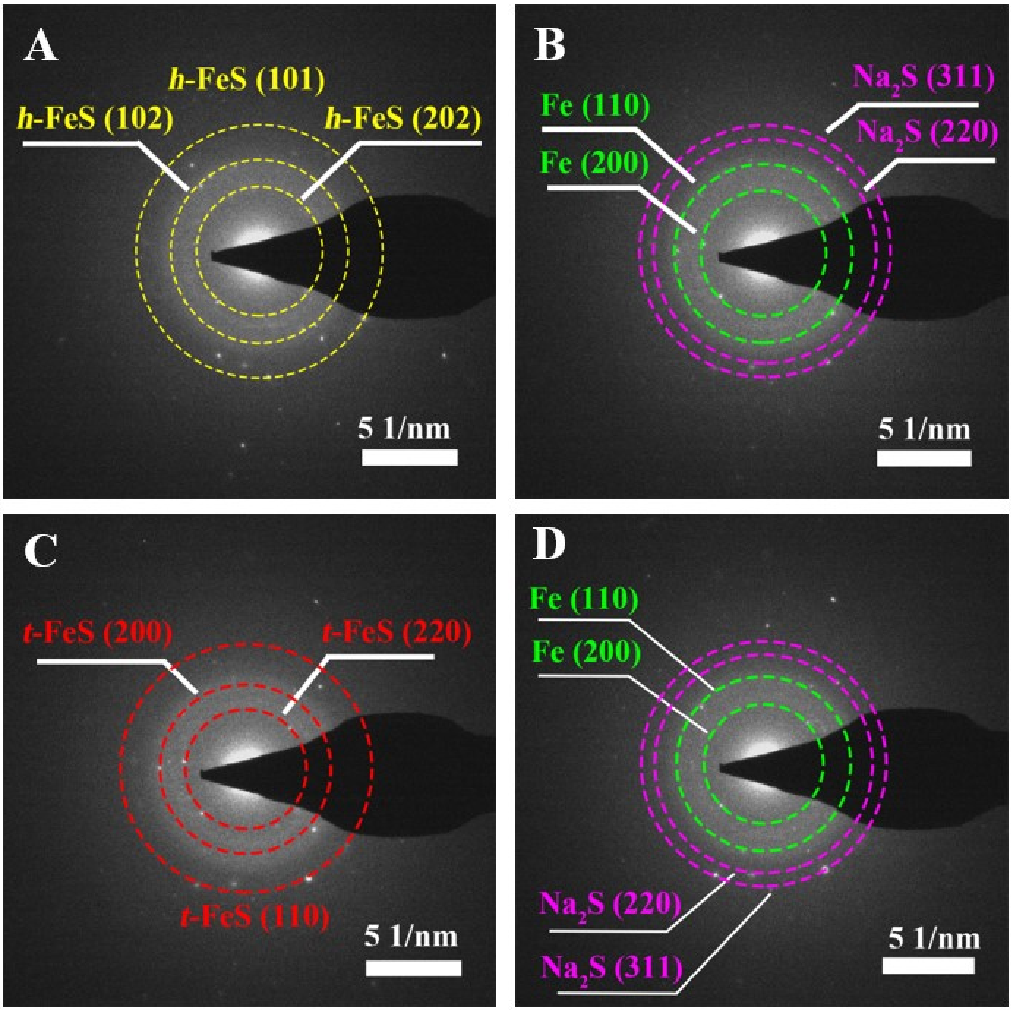

Fig. S15 SAED patterns of FeS@NSC at different sodiation/desodiation processes: (A) initial stage, (B) sodiation, (C) desodiation, and (D) sodiation.

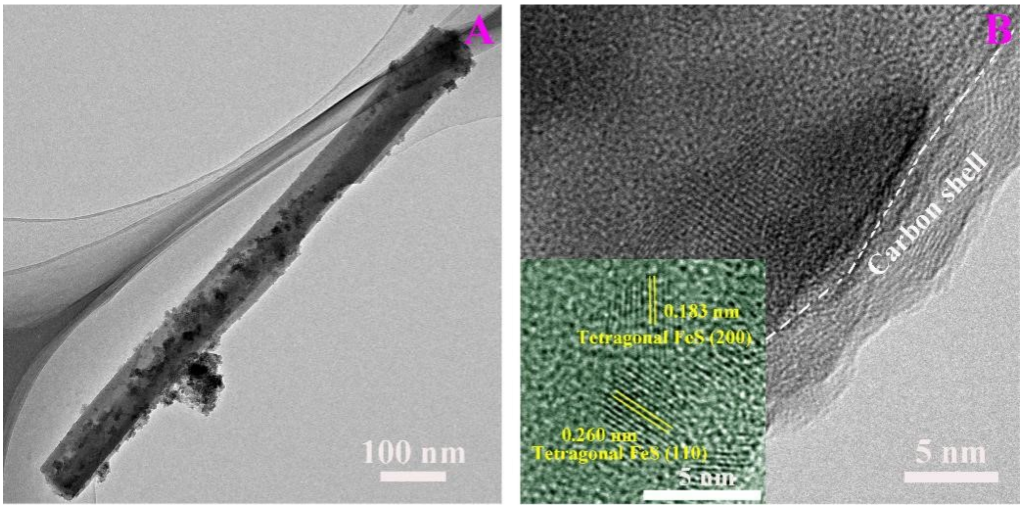

Fig. S16 (A) TEM and (B) HRTEM images of the FeS@NSC electrode charged at 3 V after 150 cycles.

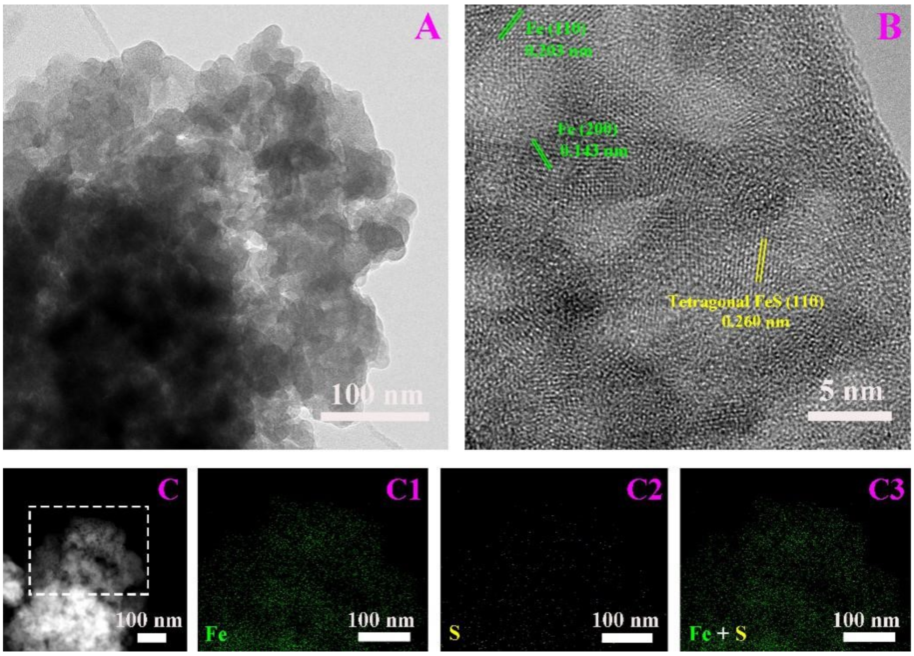

Fig. S17 (A) TEM, (B) HRTEM and (C) dark-field TEM and EDS elemental mapping images of commercial FeS electrode charged at 3 V after 150 cycles.

Table S1. Comprehensive overview of FeS-based materials recently reported in the literatures for SIB-related applications.

Metallic sulfide	Initial coulomb efficiency (%)	Discharge current (mA g^{-1})	Cycle number	Discharge capacities (2nd cycle to final cycle mAh g^{-1})	Decay capacity per cycle (mAh g^{-1})	Decay ratio per cycle (%)	Synthetic method
Ni-Fe-S-CNT [1]	62.0	100	100	864 to 431	4.33	0.501	Convenient co-precipitation + Heat treatment
Fe_7S_8/C-TiO_2 [2]	72.3	0.2C	200	588.8 to 423.3	0.828	0.141	Hydrothermal process + Thermal treatment + Sulfurization procedure
NHCFs/Fe_7S_8 [3]	89.8	1000	400	～610 to 528	0.205	0.034	Chemical bath deposition + Subsequent sulfidation treatment
Fe_7S_8@S/N-C [4]	~80.0	1000	150	～369 to ～347	0.147	0.040	Electrospinning + Sulfurization process
FeS/CFs [5]	68.9	1000	400	317 to 283	0.085	0.027	Wet-spinning process + Two-step heat treatment
US-MoS_2@NG [6]	~70.0	1000	1000	228 to 198	0.03	0.013	Solvothermal method
FeS/SPAN-HNF [7]	78	200	50	782 to 750	0.64	0.082	Electrospinning + Heat treatment
FeS@C [8]	76.5	200	150	547.1 to 555.1	-0.053	-0.009	Solvothermal method
FeS/NC [9]	51.5	200	100	599.9 to 511	0.889	0.148	Sol–gel method, Pyrolysis + Sulfidation process
S-WS_2@NC [10]	67.7	100	200	477 to 473	0.02	0.004	Solvothermal method + Heat treatment
FeS@C [11]	86.7	2000	500	413 to 407	0.012	0.003	Homogeneous carbothermal reduction strategy
FeS@NSC [This work]	68.5	100	150	611.6 to 575.0	0.244	0.039	Chemical-vapor deposition-like strategy
	71.2	10000	2000	309.3 to 273.2	0.018	0.006	
	72.4	20000	2000	261.7 to 247.8	0.007	0.003	

References

1 S. Zhang, G. Wang, B. Wang, J. Wang, J. Bai, H. Wang, *Adv. Funct. Mater.* 2020, **30**, 2001592.

2 X. Deng, H. Chen, X. Wu, Y. Wang, F. Zhong, X. Ai, H. Yang, Y. Cao, *Small*, 2020, **16**, 2000745.

3 C. Zhang, D. Wei, F. Wang, G. Zhang, J. Duan, F. Han, H. Duan, J. Liu, *J. Energy Chem.* 2021, **53**, 26-35.

4 X. Li, T. Liu, Y. Wang, S. Chou, X. Xu, A. Cao, L. Chen, *J. Power Sources*, 2020, **451**, 227790.

5 D. Li, Y. Sun, Y. Chen, J. Yao, Y. Zhang, Y. Xia, D. Yang, *ACS Appl. Mater. Interfaces*, 2018, **10**, 17175-17182.

6 X. Xu, R. Zhao, W. Ai, B. Chen, H. Du, L. Wu, H. Zhang, W. Huang, T. Yu, *Adv Mater.,* 2018, **30**, 1800658.

7 A. Haridas, J. Heo, X. Li, H. Ahn, X. Zhao, Z. Deng, M. Agostini, A. Matic, J. Ahn, *Chem. Eng. J.*, 2020, **385**, 123453.

8 B. Hou, Y. Wang, J. Guo, Q. Ning, X. Xi, W. Pang, A. Cao, X. Wang, J. Zhang, X. Wu, *Nanoscale*, 2018, **10**, 9218-9225.

9 Y. Liu, W. Zhong, C. Yang, Q. Pan, Y. Li, G. Wang, F. Zheng, X. Xiong, M. Liu, Q. Zhang, *J. Mater. Chem. A*, 2018, **6**, 24702-24708.

10 X. Hu, Y. Liu, J. Li, G. Wang, J. Chen, G. Zhong, H. Zhan, Z. Wen, *Adv. Funct. Mater.*, 2019, **30**, 1907677.

11 D. Yang, W. Chen, X. Zhang, L. Mi, C. Liu, L. Chen, X. Guan, Y. Cao, C. Shen, *J. Mater. Chem. A*, 2019, **7**, 19709-19718.
